# Alpha/Beta Interferon (IFN-α/β) Signaling in Astrocytes Mediates Protection against Viral Encephalomyelitis and Regulates IFN-γ-Dependent Responses

**DOI:** 10.1128/JVI.01901-17

**Published:** 2018-04-27

**Authors:** Mihyun Hwang, Cornelia C. Bergmann

**Affiliations:** a Department of Neurosciences, Lerner Research Institute, Cleveland Clinic Foundation, Cleveland, Ohio, USA; Loyola University Medical Center

**Keywords:** astrocytes, CNS, IFN-α, IFN-β, IFNAR, MHV

## Abstract

The contribution of distinct central nervous system (CNS) resident cells to protective alpha/beta interferon (IFN-α/β) function following viral infections is poorly understood. Based on numerous immune regulatory functions of astrocytes, we evaluated the contribution of astrocyte IFN-α/β signaling toward protection against the nonlethal glia- and neuronotropic mouse hepatitis virus (MHV) strain A59. Analysis of gene expression associated with IFN-α/β function, e.g., pattern recognition receptors (PRRs) and interferon-stimulated genes (ISGs), revealed lower basal mRNA levels in brain-derived astrocytes than in microglia. Although astrocytes poorly induced *Ifn*β mRNA following infection, they upregulated various mRNAs in the IFN-α/β pathway to a higher extent than microglia, supporting effective IFN-α/β responsiveness. Ablation of the IFN-α/β receptor (IFNAR) in astrocytes using mGFAPcre IFNAR^fl/fl^ mice resulted in severe encephalomyelitis and mortality, coincident with uncontrolled virus replication. Further, virus spread was not restricted to astrocytes but also affected microglia and neurons, despite increased and sustained *Ifn*α/β and ISG mRNA levels within the CNS. IFN-γ, a crucial mediator for MHV control, was not impaired in infected mGFAPcre IFNAR^fl/fl^ mice despite reduced T cell CNS infiltration. Unexpectedly however, poor induction of IFN-γ-dependent major histocompatibility complex (MHC) class II expression on microglia supported that defective IFN-γ signaling contributes to uncontrolled virus replication. A link between sustained elevated IFN-α/β and impaired responsiveness to IFN-γ supports the novel concept that temporally limited early IFN-α/β responses are critical for effective antiviral IFN-γ function. Overall, our results imply that IFN-α/β signaling in astrocytes is not only critical in limiting early CNS viral spread but also promotes protective antiviral IFN-γ function.

**IMPORTANCE** An antiviral state established by IFN-α/β contains initial viral spread as adaptive immunity develops. While it is apparent that the CNS lacks professional IFN-α/β producers and that resident cells have distinct abilities to elicit innate IFN-α/β responses, protective interactions between inducer and responder cells require further investigation. Infection with a glia- and neuronotropic coronavirus demonstrates that astrocytes mount a delayed but more robust response to infection than microglia, despite their lower basal mRNA levels of IFN-α/β-inducing components. Lethal, uncontrolled viral dissemination following ablation of astrocyte IFN-α/β signaling revealed the importance of IFN-α/β responses in a single cell type for protection. Sustained global IFN-α/β expression associated with uncontrolled virus did not suffice to protect neurons and further impaired responsiveness to protective IFN-γ. The results support astrocytes as critical contributors to innate immunity and the concept that limited IFN-α/β responses are critical for effective subsequent antiviral IFN-γ function.

## INTRODUCTION

Viral infections of the central nervous system (CNS) are rare but can lead to rapid mortality or long-term neurological disabilities even if acute encephalitis is resolved (1). Early essential host defense mechanisms involve induction of alpha/beta interferon (IFN-α/β) and signaling through the IFN-α/β receptor (IFNAR) to upregulate IFN-stimulated genes (ISGs). ISG expression is associated with numerous biological activities, including antiviral and immunomodulatory pathways, e.g., interference with translation, apoptosis, and enhanced major histocompatibility complex (MHC) class I antigen (Ag) presentation (2–4). While the IFN-α/β response is critical in stemming CNS viral replication and spread, it is rapidly downregulated and insufficient to eliminate virus in the absence of subsequent adaptive immune responses (5–7). Moreover, the efficiency of the innate response in limiting viral spread sets the stage for the effectiveness of subsequent adaptive immunity (2–4, 8).

Detection of viruses and induction of IFN-α/β are initiated by pattern recognition receptors (PRRs), which differ in subcellular localization and are specialized to recognize distinct molecular entities in virus structural components, genome, and/or replication intermediates (2, 8, 9). PRRs which recognize RNA viruses include endosome-associated membrane-bound Toll-like receptors (TLRs), mainly TLR3, and the cytoplasmic retinoic acid inducible gene 1 (RIG-I)-like receptors (RLRs) and melanoma differentiation-associated antigen 5 (MDA5). Specialized PRR adapter proteins transmit signals to activate IFN response factor 3 (IRF3), IRF5, and IRF7, which translocate to the nucleus to induce IFN-β and a subset of IFN-α genes (9). Secretion of IFN-α/β and signaling through IFNAR subsequently activate ISGs and the “antiviral state.” PRR activation also activates nuclear factor κB (NF-κB)-regulated genes, thereby inducing proinflammatory cytokines and chemokines to facilitate recruitment of innate and adaptive effector cells (2, 9). Virtually all nucleated cells are able to induce and respond to IFN-α/β, including CNS resident cells (5, 6). The CNS does not harbor plasmacytoid dendritic cells, which are potent peripheral IFN-α/β inducers (3, 10), and therefore has to rely on intrinsic induction of IFN-α/β. CNS resident cells have been shown to differ widely in the repertoire as well as magnitude of basal and inducible transcripts encoding PRRs and factors associated with the IFN-α/β pathway (5–7, 11). As IFN-α/β signaling can rapidly induce genes involved in virus sensing and their signaling components, cell types which are not effective initial IFN-α/β inducers may nevertheless contribute to the overall innate antiviral activity by efficient IFN-α/β signaling and amplification of the IFN-α/β response (5, 11, 12). The interdependence of CNS cells in achieving an IFN-α/β-dependent antiviral state thus requires further investigation.

Microglia and astrocytes are the CNS resident cells most prominently involved in early innate responses to injury, autoimmune attack, or infection (6, 7, 11, 12). In infections these early responses are essential to attract peripheral immune cells and limit viral dissemination (2, 3, 8). Importantly, not only productively infected but also abortively infected astrocytes mount innate responses that contribute to protection (3, 13, 14). CNS infections by several neuronotropic RNA viruses, including La Crosse virus, rabies virus, vesicular stomatitis virus (VSV), and Theiler's murine encephalomyelitis virus (TMEV), suggested that astrocytes, not neurons, are the main source of IFN-β expression and contributors to virus control through both TLR and RLR activation pathways (14–16). Both microglia and astrocytes express various PRRs, and their sentinel function is supported by their rapid response to microbial infection (5, 7, 10, 14, 17, 18). In addition to participating in direct antiviral functions, astrocytes regulate CNS homeostasis, support neuronal function, and participate in formation and maintenance of the blood-brain barrier (BBB) (19, 20). Both astrocytes and IFN-α/β actively participate in BBB function to restrict CNS entry of molecules, cells, or pathogens (19, 21). IFN-α/β signaling specifically to astrocytes promotes BBB integrity in the cerebellum and protects from West Nile virus infection (22). Thus, both astrocyte IFN-α/β induction and signaling regulate the outcome of infection within the CNS at different functional levels. The essential role of IFNAR signaling in limiting viral dissemination within the CNS is supported by neurotropic virus infections of IFNAR-deficient (IFNAR^−/−^) mice (3, 5, 6, 23). However, caveats for IFNAR^−/−^ mice are the reduced basal levels of factors involved in the IFN-α/β pathway as well as loss of innate myeloid cell dynamic activity in response to infection (11, 24, 25). Further, while much information on CNS innate responsiveness is derived from primary glial and neuronal cultures, the *in vivo* studies of IFN-β induction highlight how unsuspected players, such as abortively infected astrocytes, contribute to pathogen control (14).

Based on the prominent role of astrocytes in participating in innate responses, this study set out to assess how IFNAR ablation in astrocytes affects pathogenesis in a glia- and neuronotropic coronavirus infection. The murine coronavirus mouse hepatitis virus (MHV) strain A59 infects microglia, astrocyte, neurons, and oligodendroglia following intracranial (i.c.) administration (26, 27). Although MHVs are at best poor inducers of IFN-α/β (28–30), they do induce IFN-β in microglia/macrophages (18). Importantly, even the low levels of IFN-α/β are essential to prevent viral dissemination and mortality (31, 32). The studies here reveal distinct patterns of basal and inducible levels of mRNAs encoding components of the IFN-α/β pathway in astrocytes and microglia isolated from naive and infected adult mouse brains. Despite expressing lower baseline mRNA levels, astrocytes upregulated IFN-α/β pathway gene expression to a greater extent than microglia, supporting effective IFN-α/β responses. Ablation of IFNΑR in astrocytes using mGFAPcre IFNAR^fl/fl^ mice resulted in severe encephalomyelitis and mortality by 7 days postinfection (p.i.). This contrasted with mild clinical symptoms and no fatalities in infected control IFNAR^fl/fl^ mice. Uncontrolled viral spread throughout the CNS parenchyma of mGFAPcre IFNAR^fl/fl^ mice not only was associated with increased astrocyte infection but also affected neurons and microglia, despite overall elevated and sustained levels of mRNAs for IFN-β and IFN-α genes and ISGs. IFN-γ, a crucial mediator of MHV control in the CNS, was not impaired, despite reduced T cell CNS infiltration. Unexpectedly however, defective IFN-γ signaling was implicated by impaired induction of IFN-γ-dependent MHC class II expression on microglia. Overall our results imply that IFN-α/β signaling in astrocytes not only is critical in limiting CNS viral spread but also promotes lymphocyte-derived protective antiviral IFN-γ function.

## RESULTS

### MHV strain A59 induces type I IFN in the CNS coincident with viral replication.

**FIG 1:**
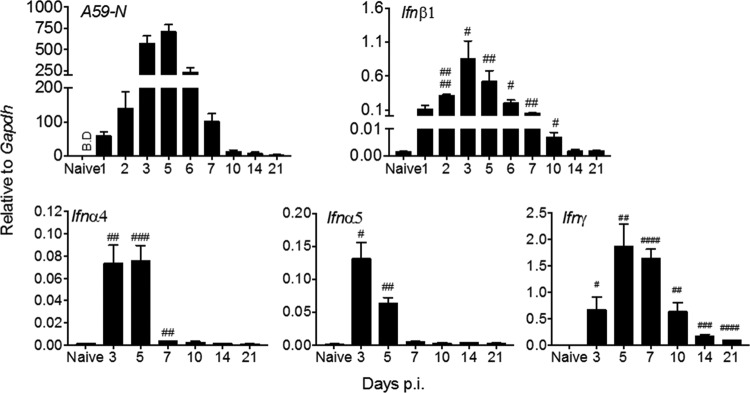
Kinetics of viral replication and induction of type I and type II IFN transcripts in the brain following MHV A59 infection. Brains from naive and infected C57BL/6 mice were harvested at indicated times p.i. and analyzed for viral nucleocapsid protein (A59-N), *Ifnβ1*, *Ifnα4*, *Ifnα5*, and *Ifn*γ mRNAs by reverse transcription-PCR (RT-PCR). Data are the means ± standard errors of the means (SEM) for *n* = 9 to 12 mice per time point from three independent experiments, each comprising 3 or 4 mice per time point, and were analyzed by the unpaired two-tailed Student *t* test and two-way ANOVA. #, significance compared to naive mice: #, *P* < 0.05; ##, *P* < 0.01; ###, *P* < 0.001; ####, *P* < 0.0001. B.D, below detection.

To evaluate the kinetics of MHV A59 replication relative to *Ifn*α/β and *Ifn*γ mRNA levels in the CNS, brains from uninfected and infected wild-type (wt) C57BL/6 mice were harvested out to day 21 p.i. Virus replication was monitored by expression of viral RNA encoding the N protein (A59 N), which is present on genomic and all subgenomic RNAs (33). Viral N mRNA was most abundant between days 3 and 5 p.i. and declined by day 7 p.i. (Fig. 1), but it remained detectable out to day 21 p.i. *Ifn*β, *Ifn*α4, and *Ifn*α5 mRNAs, the most abundant *Ifn*α mRNAs expressed following MHV A59 infection (34), also reached maximal levels between 3 and 5 days p.i. and dropped significantly by day 7 p.i. (Fig. 1). While *Ifn*β mRNA was still elevated at day 10 p.i., *Ifn*α4 and *Ifnα5* mRNAs had declined to basal levels by day 7 p.i. *Ifn*γ mRNA was already detectable at day 3 p.i. but did not peak until 5 to 7 days p.i. Expression levels declined by day 10 p.i. but remained elevated above background out to day 21 p.i. (Fig. 1). *Ifn*α/β mRNA levels thus peaked together with viral mRNA, whereas the *Ifn*γ mRNA peak was delayed and coincided with T cell infiltration (31).

### Astrocytes exhibit distinct induction of and responsiveness to IFN-α/β compared to microglia.

**FIG 2:**
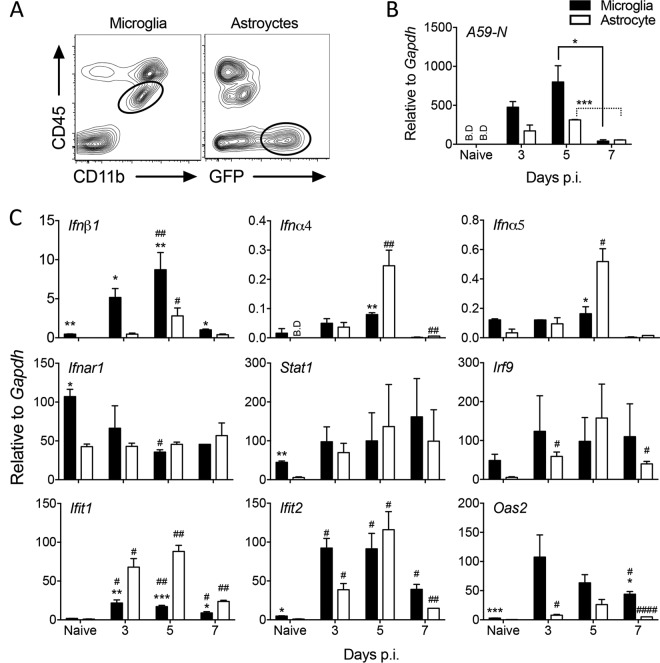
Astrocyte-mediated induction of and responsiveness to IFN-α/β compared to those for microglia. Microglia and astrocytes were isolated by FACS from brains of naive or infected GFAP-GFP mice (*n* = 5 or 6) at the indicated times p.i. based on CD45^int^ CD11b^+^ and CD45^−^ GFAP^+^, respectively. (A) Representative contour plots show the gating strategy at day 7 p.i. for microglia and astrocytes. Both plots are from total live cells. (B and C) Microglia and astrocytes were analyzed for transcripts of A59-N, *Ifnα4*, *Ifnα5*, *Ifnβ1*, *Ifnar1*, signaling components (*Stat1* and *Irf9*), and select ISGs (*Ifit1*, *Ifit2*, and *Oas2*) by RT-PCR. Data represent the means ± SEM from two experiments, each comprising 5 or 6 pooled brains per time point, and were analyzed by the unpaired two-tailed Student *t* test and two-way ANOVA. *, significance between microglia and astrocytes; #, significance compared to each respective naive population: * and #, *P* < 0.05; ** and ##, *P* < 0.01; *** and ###, *P* < 0.001; ####, *P* < 0.0001. BD, below detection. In panel B, asterisks indicate significance between days 5 and 7 p.i. in the same cell type.

Although MHV A59 replicates in glia and neurons, it induces IFN-α/β only in microglia, not astrocytes, using primary cell cultures (29). To assess the relative induction of and responsiveness to IFN-α/β in astrocytes and microglia *in vivo*, we used infected GFAP-GFP mice to isolate CD45-negative, green fluorescent protein (GFP)-positive (CD45^−^ GFP^+^) astrocytes and CD45^int^ CD11b^+^ microglia by fluorescence-activated cell sorting (FACS) (Fig. 2A). Measurement of viral N relative to glyceraldehyde-3-phosphate dehydrogenase (GAPDH) mRNAs in either glia population revealed viral replication in both microglia and astrocytes at days 3 and 5 p.i. and a decline by day 7 p.i. (Fig. 2B). While increased viral N mRNA in microglia relative to astrocytes at days 3 and 5 p.i. did not reach statistical significance, overall viral N mRNA levels mirrored those in total brain. The same populations of purified cells were used to measure transcript levels of the *Ifn*α/β genes, genes regulating IFN-α/β, signaling and selected antiviral ISGs (Fig. 2C). In naive mice, constitutive expression of these genes was lower or undetectable in astrocytes compared to microglia. Following infection, *Ifn*β mRNA was upregulated by day 3 p.i. in microglia but was not upregulated until day 5 p.i. and was less robust in astrocytes. In contrast, *Ifnα4* and *Ifnα5* mRNAs were not significantly upregulated in microglia but were increased prominently in astrocytes by day 5 p.i. Consistent with the decline in viral RNA, *Ifn*α/β mRNAs dropped to baseline levels by day 7 p.i. (Fig. 2C). Transcripts for components required for IFN-α/β signaling showed no significant differences between astrocytes and microglia throughout days 3 to 7 p.i. (Fig. 2C). While infection mediated a decline in *Ifnar1* mRNA relative to basal levels in microglia by day 5 p.i., it did not alter expression levels in astrocytes. *Stat1* mRNA levels varied between cell preparations and showed no significant changes throughout infection in either cell type. *Irf9* transcripts were not affected by virus infection in microglia but increased modestly in astrocytes. In contrast, individual ISGs were regulated distinctly not only between the glia populations but also within each glia type over time (Fig. 2C). MHV CNS infection has been shown to strongly induce IFN-induced protein with tetratricopeptide repeats (*Ifit1* and *Ifit2*) and 2′,5′-oligoadenylate synthetase 2 (*Oas2*) transcripts (11, 31). Although *Ifit1* mRNA was increased in both populations by day 3 p.i., the relative induction was significantly higher in astrocytes than in microglia at all time points. In contrast, *Ifit2* mRNA was induced more prominently in microglia at day 3 p.i., reached similar levels in both cell types at day 5 p.i., and dropped thereafter in both populations. Lastly, *Oas2* mRNA showed peak upregulation in microglia by day 3 p.i. and modest induction in astrocytes (Fig. 2C). Under the assumption that viral mRNA levels reflect similar replication in both glial populations, these data support microglia as superior initiators of IFN-β production relative to astrocytes following MHV A59 infection *in vivo*; these findings are consistent with results from primary neonatal cell cultures (18, 29). Moreover, delayed *Ifn*β upregulation in astrocytes suggested that their sensitivity to viral replication is enhanced once viral sensors are elevated via IFN-α/β signaling. This notion was supported by delayed, yet more extensive, upregulation of the *Ifn*α as well as the *Ifit1* and *Ifit2* genes compared to that in microglia. ISG upregulation further appeared to be independent of direct changes in *Ifnar*, *Stat1*, or *Irf9* transcript levels.

**FIG 3:**
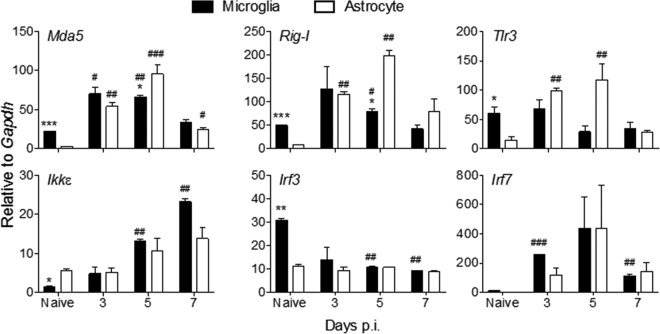
Astrocytes rapidly induce viral RNA-sensing PRRs following MHV A59 infection. Microglia and astrocytes were isolated by FACS from brains of naive or MHV A59-infected GFAP-GFP mice as indicated in Fig. 2 and analyzed for transcripts encoding RNA-sensing PRRs (*Mda5*, *Rig-I*, and *Tlr3*), kinases associated with IFN-α/β induction (*Ikk*ε), and transcription factors (*Irf3*, *Irf7*) by RT-PCR. Data represent the means ± SEM from two experiments, each comprising 5 or 6 pooled brains per time point, and were analyzed by the unpaired two-tailed Student *t* test and two-way ANOVA. *, significance between microglia and astrocytes; #, significance compared to each respective naive population: * and #, *P* < 0.05; ** and ##, *P* < 0.01; *** and ###, *P* < 0.001.

Many PRR-encoding genes are themselves ISGs and are upregulated during CNS infections (3, 11, 35, 36). MDA5, RIG-I, and TLR3 have all been established as PRRs contributing to IFN-α/β induction in MHV-infected microglia/macrophages and oligodendrocytes (18, 27, 37, 38). Basal *Mda5*, *Rig-I*, and *Tlr3* transcript levels were all significantly lower in FACS-purified astrocytes than in microglia (Fig. 3). Nevertheless, astrocytes rapidly upregulated *Mda5*, *Rig-I*, and *Tlr3* mRNAs to levels observed in microglia as early as day 3 p.i. (Fig. 3), when total *Ifn*α/β mRNA peaked in the brain (Fig. 1). Relative to basal levels, the fold increase in astrocytes exceeded that in microglia. All three PRR transcripts declined by day 7 p.i., reflecting the decline in IFN-α/β. With the exception of mRNA encoding IKKε, a kinase involved in both induction and IFNAR signaling, constitutive levels of transcripts for PRR signal transduction factors represented by IRF3 and IRF7 were also lower in astrocytes (Fig. 3). Expression of the cellular kinase *Ikk*ε mRNA was gradually increased in both glial populations over time up to day 7 p.i., with increases more pronounced in microglia (Fig. 3). *Irf3* mRNA expression was decreased in microglia following infection and remained unchanged in astrocytes, contrasting with upregulation of *Irf7* mRNA in both cell types between days 3 to 5 p.i. and downregulation thereafter (Fig. 3). These data are consistent with constitutive IRF3 expression but inducible expression of both IKKε and IRF7 following infection or IFN-α/β treatment of non-CNS cells (39). Overall, the data suggest that initial production of IFN-β in microglia results in upregulation of select ISGs in astrocytes, thereby amplifying IFN-α/β. However, it remains unclear whether astrocytes induce IFN-β directly via virus-derived PRR activators or via mediators released from necrotic or apoptotic cells in the inflamed CNS environment. Regardless, the results demonstrate that astrocytes are sensitive responders to IFN-α/β and active participants in the IFN-α/β-induced amplification loop.

### IFNAR signaling in astrocytes is essential to control MHV A59 infection.

**FIG 4:**
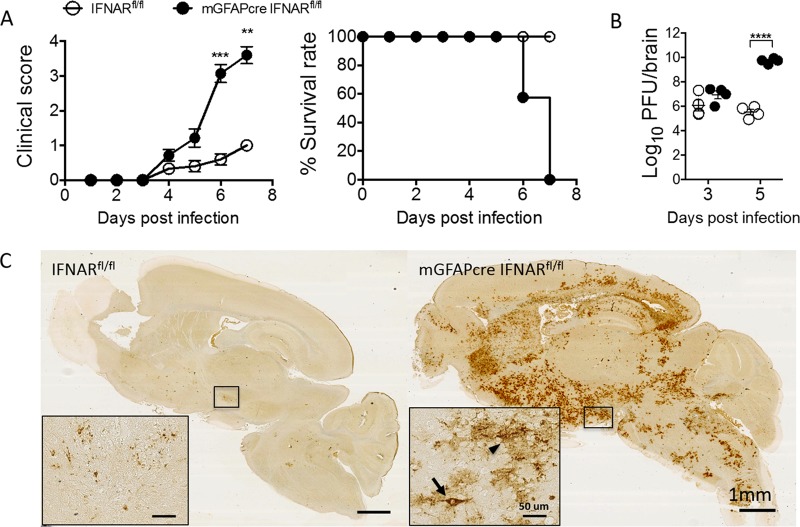
IFNAR signaling in astrocytes mediates protection against viral encephalomyelitis. (A) IFNAR^fl/fl^ (*n* = 12) and mGFAPcre IFNAR^fl/fl^ (*n* = 14) mice infected with MHV A59 were monitored for disease progression as described in Materials and Methods and for survival rate. (B) Virus titers in brain supernatants were quantified by plaque assay (*n* = 4/group/time point). Titers are from one experiment representative of two independent experiments. Data for panels A and B were analyzed by the unpaired two-tailed Student *t* test. Asterisks indicate significance between IFNAR^fl/fl^ and mGFAPcre IFNAR^fl/fl^ mice: **, *P* < 0.01; ***, *P* < 0.001; ****, *P* < 0.0001. (C) Representative images of viral N protein distribution in brains from both mouse groups at day 6 p.i. detected by immunoperoxidase staining using MAb J3.3 (red chromogen; hematoxylin counterstain). Scale bars, 1 mm. Insets show the infected area or cells at higher magnifications. The arrow indicates typical neuronal morphology, and the arrowhead indicates glial morphology. Scale bars, 50 μm.

Given the prominent response of astrocytes to IFN-α/β following MHV A59 infection *in vivo*, we determined the protective role of astrocyte-mediated IFNAR signaling against infection using mGFAPcre IFNAR^fl/fl^ mice. Disease onset was similar in mGFAPcre IFNAR^fl/fl^ and IFNAR^fl/fl^ mice starting at day 4 p.i. However, in contrast to mild clinical symptoms in IFNAR^fl/fl^ mice, mGFAPcre IFNAR^fl/fl^ mice developed severe encephalitis and succumbed to infection by day 7 p.i. (Fig. 4A). The 4- to 5-day-delayed mortality compared to that of IFNAR^−/−^ mice infected with a similar virus dose (31) supported astrocytes as critical, but not the only, contributors to early IFNAR-dependent protection.

To evaluate whether disease severity and mortality in mGFAPcre IFNAR^fl/fl^ mice were correlated with impaired viral control, infectious virus was measured in brain supernatants. The virus loads were comparable in both groups at day 3 p.i. but reached ∼5-log_10_-higher levels in mGFAPcre IFNAR^fl/fl^ mice than in IFNAR^fl/fl^ mice by day 5 p.i. (Fig. 4B). The anatomical localization and distribution of viral N protein were also evaluated by histology. Compared to small isolated residual foci of viral Ag-positive cells in brains of IFNAR^fl/fl^ mice at day 6 p.i., mGFAPcre IFNAR^fl/fl^ mice exhibited extensive dissemination with increased intensity of viral Ag staining throughout the brain (Fig. 4C). High-magnification images indicated that virus was not limited to cells consistent with glial morphology but also was in cells with typical neuronal morphology in mGFAPcre IFNAR^fl/fl^ mice (Fig. 4C, insets). As MHV A59 is hepatotropic following peripheral infection and can spread to the liver following i.c. infection of immunocompromised mice (26), we also surveyed liver for viral N mRNA. The levels of viral N relative to GAPDH transcripts were elevated in livers of mGFAPcre IFNAR^fl/fl^ compared to IFNAR^fl/fl^ mice at day 6 p.i. (values of 82 ± 48 versus 5 ± 3) (data not shown). However, overall viral N mRNA levels were significantly higher in the CNS (9,013 ± 998 versus 230 ± 55, respectively) (see Fig. 8). There was also no evidence of necrotic foci by gross visual examination (data not shown). Mortality was thus attributed to overwhelming virus spread within the CNS.

**FIG 5:**
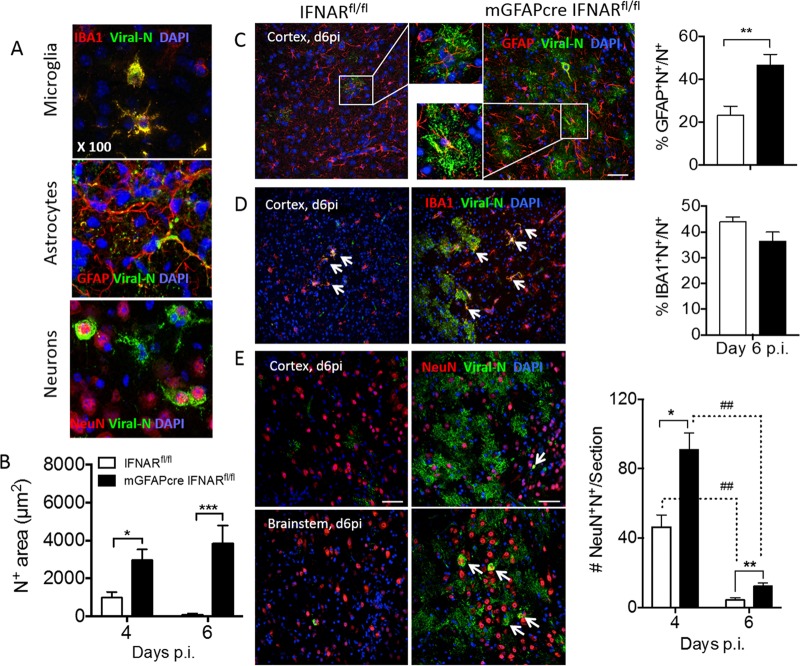
IFNAR abrogation in astrocytes results in uncontrolled virus dissemination in astrocytes and to a lesser extent in neurons and microglia. (A) Representative staining of viral N protein in various CNS cell types was determined using anti-J3.3 MAb in combination with anti-GFAP, -IBA1, or -NeuN MAb as markers for astrocytes, microglia, and neurons, respectively, and DAPI to identify nuclei. (B) Quantification of J3.3-positive area (N^+^) per field of view (μm^2^) in brains of IFNAR^fl/fl^ and mGFAPcre IFNAR^fl/fl^ mice at days 4 and 6 p.i. (C and D) Representative staining of viral N protein in astrocytes and microglia in brain cortex of both mouse groups at day 6 p.i. Bar graphs show the proportion of N^+^ GFAP^+^ or N^+^ IBA1^+^ per total N^+^ area, respectively. Insets show infected astrocytes at higher magnification and arrows indicate infected microglia. (E) Representative staining of viral N protein in neurons in brain cortex and brain stem at days 4 and 6 p.i. The bar graph shows average numbers of virus-infected neurons per brain section from 2 or 3 whole brain sections per mouse (*n* = 3 mice). Arrows indicate virus-infected neurons. Scale bar, 50 μm. (C to E) Left and right panels indicate IFNAR^fl/fl^ and mGFAPcre IFNAR^fl/fl^ mice, respectively. The data in panels B and C represent the means ± SEM from 6 or 7 fields in 2 or 3 slides from 3 mice per group and were analyzed by the unpaired two-tailed Student *t* test and two-way ANOVA. *, significance between IFNAR^fl/fl^ and mGFAPcre IFNAR^fl/fl^ mice; #, significance between days 4 and 6 p.i. in the same group: *, *P* < 0.05; ** and ##, *P* < 0.01; ***, *P* < 0.001.

**FIG 6:**
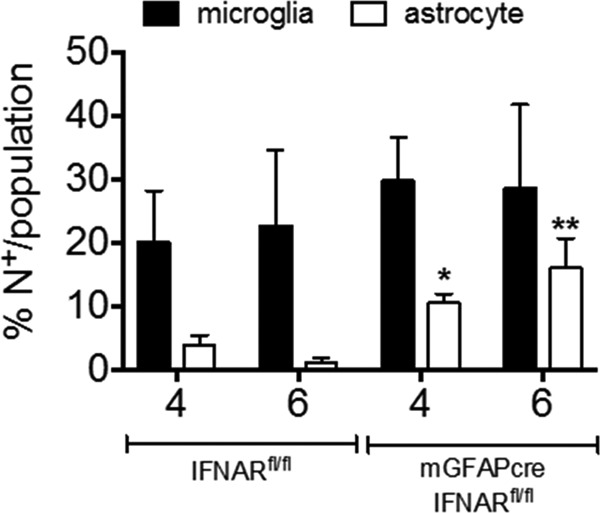
IFNAR abrogation on astrocytes predisposes astrocytes to infection over microglia. Bar graphs show the proportion of N^+^ GFAP^+^/GFAP^+^ or N^+^ IBA1^+^/IBA1^+^ per field in IFNAR^fl/fl^ (left) and mGFAPcre IFNAR^fl/fl^ (right) mice. Data represent the means ± SEM from 3 fields in 2 or 3 slides from 3 mice per group and were analyzed by the unpaired two-tailed Student *t* test. Asterisks indicate significance between IFNAR^fl/fl^ and mGFAPcre IFNAR^fl/fl^ mice, comparing the same cell populations: *, *P* < 0.05; **, *P* < 0.01.

**FIG 7:**
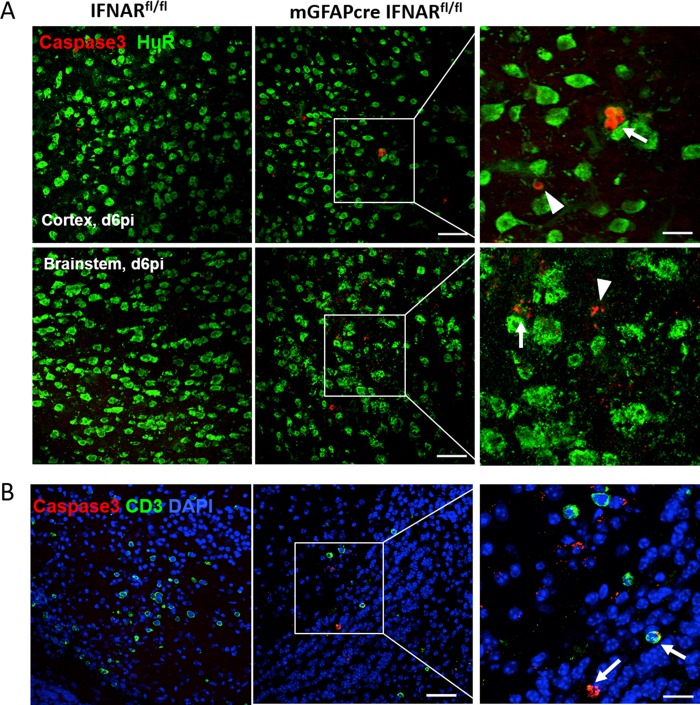
IFNAR abrogation on astrocytes enhances apoptosis of neurons and CD3^+^ cells in the CNS following infection. (A) Representative staining of apoptosis (anti-caspase 3) of neurons (anti-HuR) in brain cortex and brain stem at day 6 p.i. Arrows indicate caspase 3^+^ neurons, and arrowheads indicate nonneuronal caspase 3^+^ cells. (B) Apoptosis of T cells (anti-CD3) in mGFAPcre IFNAR^fl/fl^ mice. Arrows indicate caspase 3^+^ CD3^+^ cells. Scale bars, 50 μm and 20 μm at low and high magnifications, respectively.

MHV A59 infects multiple CNS cell types, including microglia, astrocytes, and neurons (28, 31). To evaluate to what extent the absence of IFN-α/β signaling in astrocytes predisposes astrocytes over other cell types to enhanced infection, brains from infected mGFAPcre IFNAR^fl/fl^ and IFNAR^fl/fl^ mice were analyzed for viral N protein in combination with antibodies (Abs) specific for neurons (NeuN), astrocytes (glial fibrillary acidic protein [GFAP]), and microglia/macrophages (IBA1). Characteristic staining and colocalization patterns at day 4 p.i. in wt mice are depicted in Fig. 5A. Microglia and neurons showed prominent perinuclear cytoplasmic N protein localization, with some extension into processes in microglia. In contrast, viral Ag was only occasionally detected in the cytoplasm of astrocytes and was localized mainly proximal to, but not overlapping with, astrocytic processes. In this context it is critical to note that the anti-GFAP Ab used to identify astrocytes stains main stem branches but not the cytoplasm or fine processes of astrocytes and poorly stains gray matter astrocytes (20, 40), suggesting that viral protein may localize to astrocytic processes poorly detected by anti-GFAP Ab. Total N protein staining revealed an ∼3-fold-higher positive area in mGFAPcre IFNAR^fl/fl^ than in IFNAR^fi/fi^ mice as determined by pixels per observed field at day 4 p.i.; the difference increased to ∼40-fold by day 6 p.i., when virus already declined in IFNAR^fi/fi^ mice (Fig. 5B). Keeping the limitations of GFAP staining in mind, colocalization of viral Ag with GFAP was overall increased at day 6 p.i. Furthermore, when the relative proportion of the dual-positive area within the total viral Ag-positive pixel area was assessed, percentages were similar in both mouse strains at day 4 p.i. but were 2-fold higher in mGFAPcre IFNAR^fl/fl^ mice than in controls at day 6 p.i. (Fig. 5C). Viral Ag-positive microglia were also overall increased in mGFAPcre IFNAR^fl/fl^ mice at day 6 p.i. However, the relative proportion of the dually viral Ag- and IBA1-positive area per total viral Ag-positive area was comparable between groups at both days 4 and 6 p.i. (Fig. 5D). To evaluate the relative infection of astrocytes versus microglia, we also measured the proportions of viral Ag-positive area relative to GFAP- or IBA1-positive areas. While infection of microglia increased from ∼20% to ∼30%, it increased from <5% to 15% in astrocytes in mGFAPcre IFNAR^fl/fl^ mice between days 4 and 6 p.i. (Fig. 6). Although infected neurons were increased in mGFAPcre IFNAR^fl/fl^ compared to IFNAR^fl/fl^ mice throughout the brain by day 4 p.i., they declined significantly in both groups by day 6 p.i. Remaining elevated neuronal infection in mGFAPcre IFNAR^fl/fl^ mice at day 6 p.i. was still particularly evident in lower and distal parts of the brain (pons and medulla) (Fig. 5E). Analysis of apoptosis to explain the decline in virus-infected neurons indicated overall sparse but more abundant neuronal apoptosis in mGFAPcre IFNAR^fl/fl^ mice (Fig. 7A). Occasional apoptotic CD3^+^ lymphocytes were also observed with elevated frequency in mGFAPcre IFNAR^fl/fl^ mice (Fig. 7B). While these results likely underestimate the extent of viral infection in astrocytes, they support that the absence of astrocyte IFNAR signaling leads to increased viral dissemination most prominently within astrocytes but also to microglia and neurons.

### Abrogated IFNAR signaling in astrocytes does not impair overall expression of IFN-α/β, ISGs, chemokines, and cytokines.

**FIG 8:**
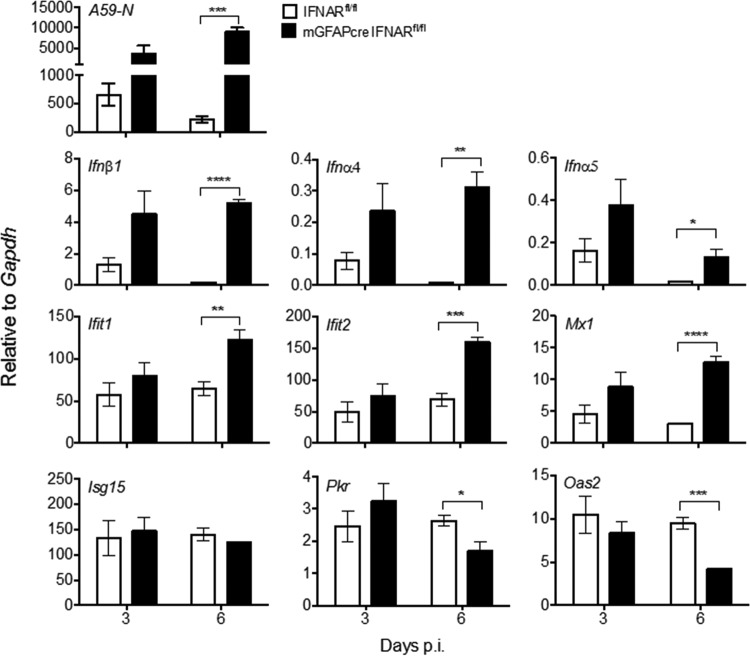
IFNAR abrogation in astrocytes results in uncontrolled viral replication and altered CNS expressions of IFN-α/β and ISGs. Brains from MHV A59-infected IFNAR^fl/fl^ and mGFAPcre IFNAR^fl/fl^ mice were analyzed for A59-N, *Ifnα4*, *Ifnα5*, *Ifnβ1*, and ISG (*Ifit1*, *Ifit2*, *Mx1*, *Isg15*, *Oas2*, and *Pkr*) mRNA levels by quantitative RT-PCR. Data represent the means ± SEM for *n* = 4 mice per time point from one experiment representative of two independent experiments and were analyzed by the unpaired two-tailed Student *t* test. Asterisks indicate significance between IFNAR^fl/fl^ and mGFAPcre IFNAR^fl/fl^ mice: *, *P* < 0.05; **, *P* < 0.01; ***, *P* < 0.001; ****, *P* < 0.0001.

To assess whether uncontrolled virus spread was attributable to overall impaired innate immune responses, we determined transcripts for IFN-α/β and ISGs in relation to viral N mRNA levels in brains of both mouse groups (Fig. 8). Viral N mRNA levels were similar at day 3 p.i. but increased significantly in mGFAPcre IFNAR^fl/fl^ mice compared to IFNAR^fl/fl^ controls by day 6 p.i. (Fig. 8), consistent with increased infectious virus by day 5 p.i. (Fig. 4B). Elevated viral N mRNA correlated with increased *Ifn*β, *Ifnα4*, and *Ifnα5* transcripts at day 6 p.i. (Fig. 8). To assess how elevated IFN-α/β affected downstream IFNAR signaling, we further compared mRNA levels of the ISGs (*Ifit1*, *Ifit2*, *Isg15*, *Mx1*, *Pkr*, and *Oas2*). While these transcripts were all similar in both mouse groups at day 3 p.i., *Ifit1*, *Ifit2*, and *Mx1* mRNAs were increased 2- to 3-fold in mGFAPcre IFNAR^fl/fl^ mice by day 6 p.i. *Isg15*, *Pkr*, and *Oas2* mRNAs were not affected or reduced relative to those in IFNAR^fl/fl^ controls (Fig. 8). IFNAR abrogation on astrocytes thus did not impair global *Ifn*α/β or select ISG expression in the infected CNS. However, innate immunity by IFNAR-competent cells was clearly insufficient to stem viral dissemination throughout the CNS in mGFAPcre IFNAR^fl/fl^ mice.

**FIG 9:**
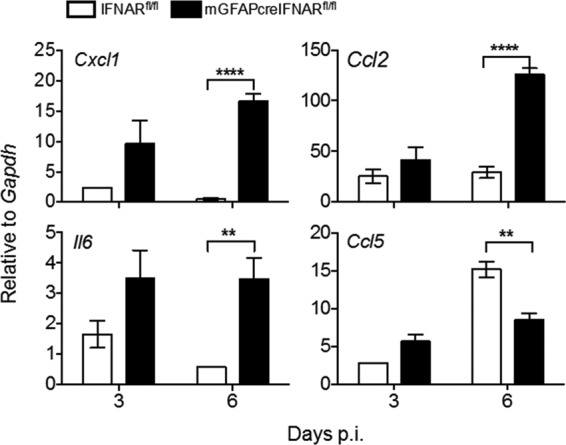
Abrogated IFNAR signaling in astrocytes leads to increased expression of proinflammatory factors. Brains from MHV A59-infected IFNAR^fl/fl^ and mGFAPcre IFNAR^fl/fl^ were analyzed for *Cxcl1*, *Ccl2*, *Ccl5*, and *Il6* mRNA levels by quantitative RT-PCR. Data represent the means ± SEM for *n* = 4 mice per time point from one experiment representative of two independent experiments and were analyzed by the unpaired two-tailed Student *t* test. Asterisks indicate significance between IFNAR^fl/fl^ and mGFAPcre IFNAR^fl/fl^ mice: **, *P* < 0.01; ****, *P* < 0.0001.

Astrocytes are also potent inducers of chemokines and cytokines (13, 41), and a subset of chemokines, e.g., the neutrophil chemoattractant CXCL1, can be negatively regulated by IFN-α/β (42). We therefore evaluated how the absence of IFNAR on astrocytes modulates expression of select NF-κB-dependent chemokines and the proinflammatory cytokine interleukin 6 (IL-6) (43, 44). Transcripts for *Cxcl1* and *Ccl2*, potent chemoattractants for neutrophils and monocytes, respectively, were similar at day 3 p.i. but were significantly upregulated at day 6 p.i. (Fig. 9), consistent with increased viral replication and IFN-α/β-mediated downregulation of CXCL1 by astrocytes (42, 45). Increased *Il6* mRNA expression at day 6 p.i. (Fig. 9) further indicated that IFNAR abrogation on astrocytes does not impact proinflammatory cytokine induction. In contrast, *Ccl5* mRNA, which is prominently expressed by T cells during MHV infection (46), was significantly reduced compared to that in IFNAR^fl/fl^ mice at day 6 p.i. Dysregulation of these proinflammatory factors suggested that myeloid cells may be recruited in favor of lymphocytes in mGFAPcre IFNAR^fl/fl^ mice, thereby contributing to morbidity and uncontrolled viral spread.

### Infected mGFAPcre IFNAR^fl/fl^ mice exhibit increased CNS neutrophil accumulation and reduced T cell infiltration.

**FIG 10:**
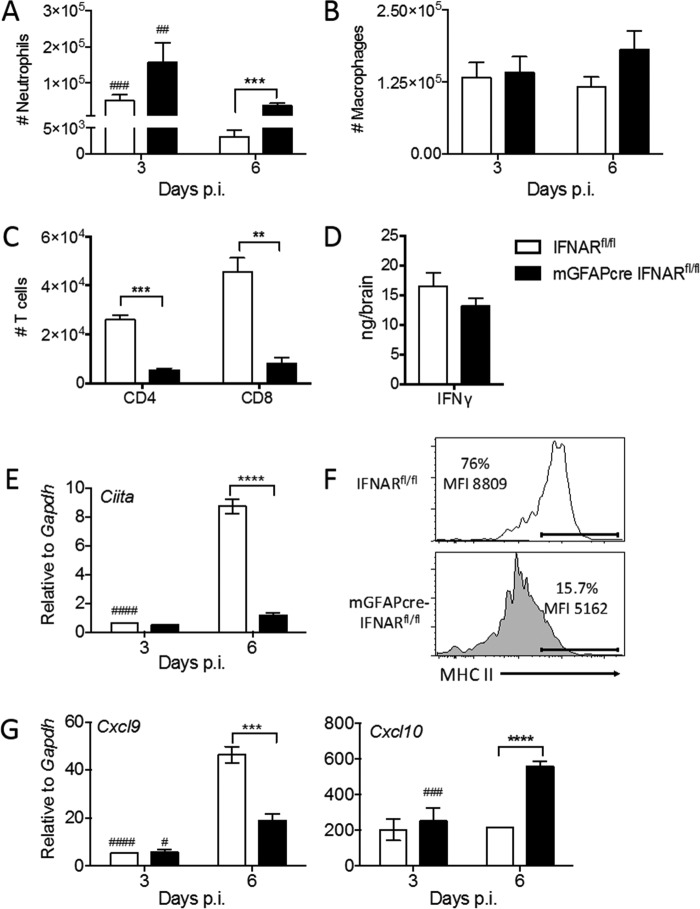
Abrogated IFNAR signaling in astrocytes is associated with altered leukocyte accumulation and impaired IFN-γ signaling in microglia. (A to C) Numbers of infiltrated neutrophils (A), macrophages (B), and CD4 and CD8 T cells (C) within brains of infected IFNAR^fl/fl^ and mGFAPcre IFNAR^fl/fl^ mice determined by flow cytometry at the indicated time points. (D) IFN-γ protein in brain supernatants measured by ELISA. (E) *Ciita* mRNA levels in brains determined by quantitative RT-PCR at days 3 and 6 p.i. (F) MHC class II expression (percentages and mean fluorescence intensity [MFI]) on microglia in brains of both mouse groups determined by flow cytometry at day 6 p.i. (G) *Cxcl9* and *Cxcl10* mRNA levels in brains measured by quantitative RT-PCR. Data represent the means ± SEM from three experiments (*n* = 9 to 12 per group and time point) and were analyzed by the unpaired two-tailed Student *t* test and two-way ANOVA. *, significance between IFNAR^fl/fl^ and mGFAPcre IFNAR^fl/fl^ mice; #, significance between days 3 and 6 p.i. in the same group: #, *P* < 0.05; ** and ##, *P* < 0.01; *** and ###, *P* < 0.001; **** and ####, *P* < 0.0001.

Flow cytometry revealed that altered chemokine expression patterns indeed affected the composition of inflammatory cells within the CNS. Consistent with elevated *Cxcl1* mRNA, neutrophils in mGFAPcre IFNAR^fl/fl^ brains were increased by ∼8-fold at day 6 p.i. relative to those in IFNAR^fl/fl^ mice, although numbers in both groups were already reduced compared to those at day 3 p.i. (Fig. 10A). Differences in macrophages were not statistically significant (Fig. 10B). As both CD4 and CD8 T cells are essential to reduce infectious MHV, with IFN-γ playing a prominent antiviral role (33, 47), we further assessed if impaired T cell recruitment and IFN-γ production within the CNS is associated with lack of virus control. Indeed, both CD4 and CD8 T cells were reduced in the CNS of mGFAPcre IFNAR^fl/fl^ mice at day 6 p.i. (Fig. 10C). However, similar IFN-γ levels (Fig. 10D), despite reduced T cell numbers, suggested that increased viral replication leads to increased Ag presentation, thereby triggering more or prolonged IFN-γ production by T cells in mGFAPcre IFNAR^fl/fl^ mice (48, 49). Impaired access of T cells to the CNS parenchyma was ruled out as an explanation of loss of viral control, as we found no differences in anatomical distribution of CD3^+^ T cells within perivascular and parenchymal sites between the two groups (data not shown). Uncontrolled viral replication in mGFAPcre IFNAR^fl/fl^ mice despite similar IFN-γ production thus implied that viral replication overwhelms the T cell response or that IFN-γ signaling is impaired.

Determination of IFN-γ-specific responses *in vivo* is complicated by the fact that many factors upregulated by IFN-γ are also induced by IFN-α/β (50). Nevertheless, a prominent indicator of IFN-γ signaling in microglia/macrophages is induction of the transcription factor class II transactivator (CIITA), a master regulator of MHC class II expression (51, 52). Interestingly, *Ciita* transcripts were barely induced in brains of mGFAPcre IFNAR^fl/fl^ mice, in contrast to robust upregulation in control mice, at day 6 p.i. (Fig. 10E). Analysis of MHC class II expression on microglia by flow cytometry confirmed that the very modest *Ciita* mRNA induction impaired IFN-γ-dependent MHC class II upregulation. Only ∼16% of microglia expressed MHC class II in mGFAPcre IFNAR^fl/fl^ mice, compared to ∼76% in IFNAR^fl/fl^ mice, and the mean fluorescence intensity, reflecting expression levels, per cell was also lower (Fig. 10F).

Other IFN-γ-dependent factors include the chemokines CXCL9 and CXCL10 (53, 54). While CXCL9 expression is inducible mainly by IFN-γ, CXCL10 is inducible by IFN-α/β, IFN-γ, and tumor necrosis factor (TNF) (42, 55, 56). Furthermore, CXCL9 is produced by macrophages/microglia and endothelial cells, but not astrocytes, whereas CXCL10 is induced prominently in astrocytes during inflammation (53, 57). Assessment of *Cxcl9* and *Cxcl10* mRNAs showed similarly low levels in both groups at day 3 p.i. (Fig. 10G). However, unlike IFNAR^fl/fl^ mice, mGFAPcre IFNAR^fl/fl^ mice failed to upregulate *Cxcl9* mRNA by day 6 p.i. In contrast, *Cxcl10* mRNA levels were elevated by ∼2.5-fold in mGFAPcre IFNAR^fl/fl^ mice by day 6 p.i. but did not increase in IFNAR^fl/fl^ mice. Given that mGFAPcre IFNAR^fl/fl^ mice do not express IFNAR on astrocytes, *Cxcl10* mRNA upregulation is likely driven by IFN-γ and/or TNF. Overall, these results implied that microglia, but not astrocytes, are significantly compromised in IFN-γ responsiveness in mGFAPcre IFNAR^fl/fl^ mice.

### Preexposure to IFN-α/β modifies IFN-γ responsiveness in myeloid cells.

**FIG 11:**
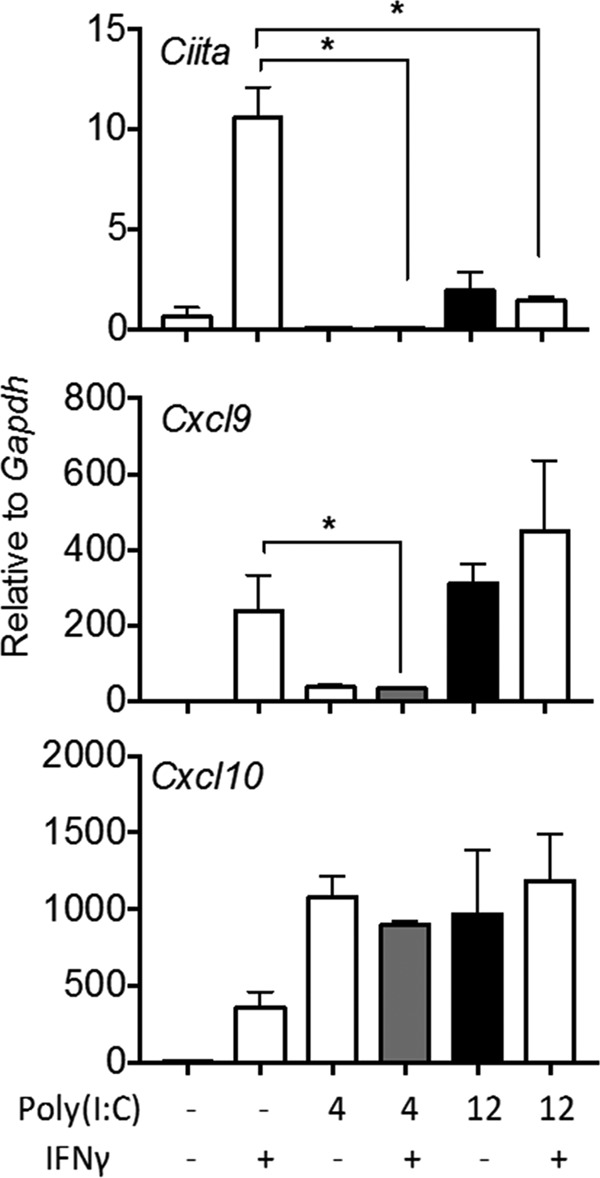
Poly(I·C) pretreatment impairs IFN-γ signaling in macrophages. Cultured BMDM were either treated with IFN-γ only (50 ng/ml) for 18 h or pretreated with poly(I·C) (5 μg/ml) for 4 or 12 h prior to treatment with IFN-γ for 18 h. *Ciita*, *Cxcl9*, and *Cxcl10* mRNA levels were determined by quantitative real-time PCR in control and treated cultures as indicated. Data represent the means ± SEM from three independent experiments. *, *P* < 0.05 compared to IFN-γ-only treatment.

One explanation for the differential responsiveness to IFN-γ in microglia versus astrocytes in mGFAPcre IFNAR^fl/fl^ mice is that prolonged IFNAR signaling via elevated and sustained IFN-α/β expression may skew subsequent IFN-γ responsiveness. This is supported by an antagonistic effect of IFN-α/β on IFN-γ responses in macrophages following IFN treatment or Listeria infection (58–60). We therefore used the viral RNA mimic poly(I·C), a synthetic double-stranded RNA (dsRNA) analog recognized by TLR3 as well as RIG-I/MDA5, to induce *Ifn*α/β mRNA in bone marrow-derived macrophages (BMDM) and monitor IFN-γ responsiveness. Previous *in vivo* data revealed that poly(I·C) administration into the CNS of mice induced *Ifn*α/β mRNA by 4 h, which was sustained out to 12 h (11). BMDM were thus pretreated with poly(I·C) for 4 or 12 h and subsequently treated with IFN-γ for 18 h. IFN-γ-only treatment was included as a positive control. Poly(I·C) alone did not induce *Ciita* mRNA at 4 h and induced it only sparsely by 12 h. However, while IFN-γ treatment alone effectively induced *Ciita* mRNA, it failed to induce *Ciita* mRNA in cells pretreated with poly(I·C) for either 4 or 12 h (Fig. 11). Similarly, poly(I·C) alone only sparsely induced *Cxcl9* mRNA at 4 h compared to induction by IFN-γ treatment alone, and short poly(I·C) pretreatment suppressed *Cxcl9* mRNA induction by IFN-γ. Assessment of an inhibitory effect of prolonged poly(I·C) exposure was preempted by induction of *Cxcl9* mRNA levels similar to those with IFN-γ. Finally, treatment with poly(I·C) alone was superior to IFN-γ alone in upregulating *Cxcl10* mRNA, with saturation already achieved at 4 h. These results are consistent with poly(I·C)-mediated upregulation of CXCL10 via IFN-α/β and TNF and preempted use of *Cxcl10* as a target gene to assess suppressive functions on IFN-γ signaling in this experimental setting. Overall, these data indicate that preexposure of macrophages/microglia to a virus-induced inflammatory milieu including IFN-α/β has the potential to negatively regulate subsequent IFN-γ signaling. However, the magnitude of IFN-α/β-mediated suppression of IFN-γ-dependent gene induction depends on other regulatory elements in the respective target gene promoter. These results reveal that the duration and/or level of cell exposure to IFN-α/β can substantially diminish responsiveness to temporally lagging IFN-γ, thereby reducing IFN-γ protective functions.

## DISCUSSION

The ability to induce and respond to IFN-α/β varies between cell types as well as between types of virus, but the contribution of different CNS cell types to mount IFN-α/β-mediated protection *in vivo* are less well explored (6). Our studies comparing the capacity of adult astrocytes relative to microglia to respond to MHV A59 infection revealed that astrocytes expressed lower basal levels of PRRs and signaling components to induce IFN-α/β and ISGs than microglia, consistent with published gene array data (61). Nevertheless, despite a delay in response to infection, astrocytes exhibited strong upregulation of PRRs, *Ifn*αs, and select ISGs, supporting that astrocytes are well positioned to respond to paracrine IFN-α/β to amplify their innate antiviral response. The critical role of IFNAR signaling in astrocytes to block viral spread was confirmed by rapid virus dissemination throughout the CNS upon abrogation of IFNAR signaling in astrocytes. Thus, although basal expression levels of IFN-α/β signaling components have been shown to determine the responsiveness of cells to insults (11, 62), our data reveal that cells expressing low basal levels of IFN-α/β pathway components can nevertheless trigger a protective innate immune response through paracrine IFN-α/β signaling.

Delayed mortality of infected mGFAPcre IFNAR^fl/fl^ mice compared to IFNAR^−/−^ mice implied that other cell types, including microglia, can produce and respond to IFN-α/β. This is clearly demonstrated by elevated and sustained *Ifn*α/β as well as select ISG expression despite IFNAR deletion on astrocytes. However, vast dissemination of virus throughout the CNS clearly indicated that IFNAR signaling specifically in astrocytes plays a dominant role in host protection, which could not be compensated by IFNAR-competent glia and neurons. While microglia appeared to exhibit some autocrine action as suggested by less overall infection relative to that in astrocytes, neurons in the brain stem appeared to be most susceptible and vulnerable to infection in the absence of astrocyte IFNAR signaling. Although we cannot exclude that inherently reduced activation of innate viral components in neurons may contribute to their susceptibility, our data indicate that overwhelming infection of IFNAR-deficient astrocytes increases adjacent neuronal infection. West Nile virus infection of neuronal cultures demonstrates that neurons have the capacity to mount innate viral responses via TLR and IRF activations (63, 64). Moreover, neurons have also been demonstrated to induce IFN-β following TMEV and La Crosse virus infection *in vivo* (65). Nevertheless, these responses were very sparse and focal. In contrast, subsequent studies of La Crosse virus infection in IFN-β reporter mice showed that apparently noninfected astrocytes and microglia in close proximity to infected neurons were prominent IFN-β-producing cells, while IFN-β expression by infected neurons was rare (16). More recent data demonstrate that IFN-β-producing astrocytes appeared to be transiently or abortively infected during various infections associated with neuronal tropism (14). The spatial extent of innate protection induced in an infected environment may thus depend on the prominent infected cell type. As both microglia and astrocytes have far-reaching processes, release of IFN-α/β from these cells may provide more extensive protection than release from neuronal cell bodies. Two independent studies with VSV indeed indicate that locally induced IFN-β in the olfactory bulb can trigger ISGs in distal parts of the brain, although one indicates astrocytes and the other neurons as the prominent IFN-β inducers (15, 66). Regardless, the vast increase of virus in mGFAPcre IFNAR^fl/fl^ mice supports the notion that the inability of astrocytes to clear virus, rather than loss of direct ISG effects on other CNS cells, is responsible for loss of viral control.

In addition to uncontrolled virus, elevated neutrophil accumulation may contribute to tissue damage and rapid morbidity of mGFAPcre IFNAR^fl/fl^ mice. Increased neutrophil CNS infiltration correlated with increased mRNA expression of the neutrophil chemoattractant CXCL1 (41) and its downregulation by IFN-α/β and IFN-γ (42, 45, 67, 68). Neutrophils are early innate responders, which contribute to BBB breakdown and tissue damage via secretion of matrix metalloproteinases (MMPs) and a wide range of other degrading enzymes released upon degranulation (69, 70). During autoimmune-mediated CNS inflammation, neutrophils have also been shown to release cytokines which mature antigen-presenting cells and subsequently reactivate T cells (71–73). Elevated *Cxcl1* mRNA as well as increased levels of mRNAs encoding IL-6 and CCL2, two other proinflammatory factors prominently induced in astrocytes (74), further suggested that the NF-κB pathway initiated by PPR activation was intact in infected mGFAPcre IFNAR^fl/fl^ mice.

Finally, our results show that extensive virus spread in the absence of protective astrocyte IFNAR signaling cannot be controlled by T cells. Although previous studies of CXCL9 and CXCL10 monoclonal antibody (MAb) blockade to reduce CXCR3-dependent T cell recruitment showed a reduction in T cell migration and loss of viral control in a related MHV infection (75, 76), subsequent studies in CXCL9- and CXCL10-deficient mice indicated that CXCL10 is more critical for effective adaptive immunity (57). Impaired T cell accumulation despite unimpaired *Cxcl10* mRNA in mGFAPcre IFNAR^fl/fl^ mice was thus unanticipated. While an overwhelming virus load may lead to Ag-induced T cell exhaustion and death (77), dysregulation of T cells requires future investigation. Regardless of reduced T cell numbers, their initial activity as monitored by IFN-γ secretion was not impaired. IFN-γ signaling is essential for MHV control within the CNS by both exerting direct antiviral effects and enhancing MHC class I and II upregulation (78). However, reduced induction of IFN-γ-dependent *Ciita* and *Cxcl9* mRNAs showed that microglia were significantly impaired in IFN-γ responsiveness. In contrast, increased *Cxcl10* mRNA expression in the absence of IFNAR on astrocytes implied that IFN-γ signaling in astrocytes remained intact. The notion that elevated and sustained IFN-α/β signaling acts as a negative regulator of IFN-γ signaling was supported by earlier reports showing antagonistic effects of IFN-α/β on IFN-γ-induced activation of macrophages (58–60). One possible mechanism may involve downregulation of the IFN-γ receptor (IFNGR), as shown during Listeria infection (58). Our *in vitro* studies further supported selected downregulation of IFN-γ responsiveness by IFN-α/β via pretreatment of BMDM with the dsRNA mimic poly(I·C). IFN-α/β-dependent sensitization of microglia/macrophages to subsequent IFN-γ responses may provide a mechanism to limit excessive immune responses leading to tissue damage. In this context it is of interest to note that both IFN-α/β and IFN-γ responses are tightly controlled by negative regulators, including phosphatases (79). Cross talk between IFN-α/β and IFN-γ responses may thus provide additional levels to protect the host from exacerbated proinflammatory responses.

Overall, our data demonstrate a vital protective role of astrocyte-mediated IFN-α/β signaling in host protection from neurotropic coronavirus-induced encephalomyelitis. Further, the link between sustained elevated IFN-α/β and impaired responsiveness to IFN-γ supports the novel concept that temporally limited early IFN-α/β responses are critical for effective antiviral IFN-γ function.

## MATERIALS AND METHODS

### Mice, virus, and infection.

FVB/N-Tg (GFAP-GFP) 14 Mes/J (GFAP-GFP) mice were originally purchased from the Jackson Laboratory (Bar Harbor, ME) and backcrossed to C57BL/6 mice for at least 10 generations. B6.Cg-Tg (Gfap-cre) 77.6Mvs/J (stock number 012887; mGFAPcre) mice were also purchased from the Jackson Laboratory (Bar Harbor, ME). IFNAR^fl/fl^ mice engineered to contain *loxP* sites flanking exon 10 of the IFNAR gene were originally produced on the 129/Ola background in U. Kalinke's laboratory (Paul-Ehrlich-Institut, Langen, Germany) as described previously (80) and were backcrossed onto the C57BL/6 background in R. Schreiber's laboratory (Washington University School of Medicine, St. Louis, MO) as described previously (81). IFNAR^fl/fl^ mice were crossed with mGFAPcre^+/−^ mice to generate mGFAPcre^+/−^ IFNAR^fl/fl^ (mGFAPcre IFNAR^fl/fl^) mice, which were crossed with IFNAR^fl/fl^ mice for experimentation. Offspring were genotyped for both the presence of the Cre recombinase gene and exon 10 deletion from the IFNAR gene by PCR using the following primers: Cre, 5′-GTCCAATTTACTGACCGTACACC-3′ (forward) and 5′-GTTATTCGGATCATCAGCTACACC-3′ (reverse); IFNAR flox, 5′-TGCTTTGAGGAGCGTCTGGA-3′ (forward) and 5′-CATGCACTACCACACCAGGCTTC-3′ (reverse). Cre-negative IFNAR^fl/fl^ littermates were used as wt controls (IFNAR^fl/fl^). All mice were housed under specific-pathogen-free conditions at an accredited facility at the Cleveland Clinic Lerner Research Institute. Mice of both sexes were infected intracranially (i.c.) at 6 to 7 weeks of age with 2,000 PFU of the hepato- and neurotropic MHV A59 strain expressing enhanced green fluorescent protein (EGFP), kindly provided by Volker Thiel (Kantonal Hospital, St. Gallen, Switzerland) (82). MHV A59 was propagated on delayed brain tumor (DBT) astrocytoma monolayers, and virus titers were determined as described previously (83). Virus in the CNSs of individual mice was measured by plaque assay on DBT cells using clarified supernatants from brain homogenates prepared as described previously (84). Clinical disease severity was graded daily using the following scale: 0, no disease symptoms; 1, ruffled fur; 2, hunched back or inability to turn upright; 3, severe hunching/wasting or hind limb paralysis; 4, moribund condition or death (31). All animal procedures were approved by the Institutional Animal Care and Use Committee of the Cleveland Clinic (PHS assurance number A3047-01) and were conducted in compliance with the Guide for the Care and Use of Laboratory Animals from the National Research Council.

### Isolation of CNS cells and flow cytometric analysis.

Brains from mice perfused with cold phosphate-buffered saline (PBS) were homogenized in Dulbecco's PBS (DPBS) (pH 7.4) using Tenbroeck tissue homogenizers as described previously (84). Homogenates were centrifuged at 450 × *g* for 10 min at 4°C. Cells were resuspended in RPMI containing 25 mM HEPES (pH 7.2), adjusted to 30% Percoll (Pharmacia, Uppsala, Sweden), underlaid with 1 ml 70% Percoll, and centrifuged at 850 × *g* for 30 min at 4°C. Cells were collected from the 30%/70% interface, washed with RPMI, counted, and suspended in fluorescence-activated cell sorting (FACS) buffer (0.1% bovine serum albumin in DPBS). Fcγ receptors were blocked with 1% mouse serum and rat anti-mouse CD16/32 MAb (clone 2.4G2: BD Biosciences, San Diego, CA) for 20 min on ice prior to staining with fluorescein isothiocyanate (FITC)-, phycoerythrin (PE)-, peridinin chlorophyll protein (PerCP)-, or allophycocyanin (APC)-conjugated MAbs specific for CD45 (clone 30-F11), CD8 (clone 53-6.7), CD4 (clone GK1.5), Ly6G (clone 1A8), CD11b (clone M1/70), and MHC class II (clone M5/114.15.2) (all from BD Bioscience, Mountain View, CA) and F4/80 (Serotec, Raleigh, NC) in FACS buffer. Samples were analyzed on a BD Accuri C6 Plus instrument (BD Biosciences). Forward- and side-scatter signals obtained in linear mode were used to establish a gate containing live cells while excluding dead cells and tissue debris. Data were analyzed using FlowJo 9 software (Tress Star Inc., Ashland, OR).

### Microglia and astrocyte isolation for gene expression analysis.

Brains from 5 or 6 naive or MHV A59-infected GFAP-GFP mice at days 3, 5, and 7 p.i. were homogenized using a neural tissue dissociation kit (Papain, Miltenyi Auburn, CA) following the manufacturer's protocol. Brain homogenates were prepared for 30/70% Percoll gradients to isolate cells as described above. CNS cells were blocked with mouse serum and anti-CD16/32 MAb as described above prior to staining with CD45 and CD11b in FACS buffer. Microglia and astrocytes were sorted based on their CD45^int^ CD11b^+^ and CD45^neg^ GFP^+^ phenotypes, respectively, using a FACS Aria III (BD Biosciences) and FACS Diva software (BD Biosciences). Sorted cells were resuspended in TRIzol (Invitrogen, Carlsbad, CA) and stored at −80°C.

### Gene expression analysis.

RNA from brains or sorted cells was extracted using TRIzol reagent (Invitrogen, Carlsbad, CA) according to the manufacturer's instructions. Following treatment with DNase I using a DNA-free kit (Ambion, Austin, TX), cDNA was synthesized using Moloney murine leukemia virus reverse transcriptase (Invitrogen) in buffer containing 10 mM deoxynucleoside triphosphate mix, 250 ng random hexamer primers, and oligo(dT) (1:1 ratio) (Invitrogen). RNA expression was assessed using either SYBR green master mix (Applied Biosystems, Foster City, CA) or TaqMan fast master mix (Applied Biosystems, Foster City, CA) as described previously (31). The following primers were used with SYBR green master mix: for the GAPDH gene, 5′-CATGGCCTTCCGTGTTCCTA-3′ (forward) and 5′-ATGCCTGCTTCACCACCTTCT-3′ (reverse); for *Cxcl9*, 5′-TGCACGATGCTCCTGCA-3′ (forward) and 5′-AGGTCTTTGAGGGATTTGTAGTGG-3′ (reverse); for *Cxcl10*, 5′-GACGGTCCGCTGCAACTG-3′ (forward) and 5′-GCTTCCCTATGGCCCTCATT-3′ (reverse); for the viral nucleocapsid (N) gene, 5′-GCCAAATAATCGCGCTAGAA-3′ (forward) and 5′-CCGAGCTTAGCCAAAACAAG-3′ (reverse); for *Il6*, *5′*-ACACATGTTCTCTGGGAAATCGT-*3′* (forward) and 5′-AAGTGCATCATCGTTGTTCATACA-3′ (reverse); for *Oas2*, 5′-AAAAATGTCTGCTTCTTGAATTCTGA-3′ (forward) and 5′-TGTGCCTTTGGCAGTGGAT-3′ (reverse); for *Ifnar1*, 5′-CCCAAGGCAAGAGCTATGTC-3′ (forward) and 5′-TCTGAACGGCTTCCAGAACT-3′ (reverse); for *Ikkε*, 5′-CCAGAAGATTCAGTGTTGTTTGG-3′ (forward) and 5′-TCATTGTAGCTGAGCCCTG-3′ (reverse); for *Mda5*, 5′-GACACCAGAATTCAAGGGAC-3′ (forward) and 5′-GCCACACTTGCAGATAATCTC-3′ (reverse); and for *Rig-I*, 5′-GTCAGCACAAACCACAACC-3′ (forward) and 5′-GTCTCAACCACTCGAATGTC-3′ (reverse). TaqMan fast master mix and TaqMan primers/probes were used to assess GAPDH, *Ifnα4*, *Ifnα5*, *Ifnβ1*, *Ifit1*, *Ifit2*, *Mx1*, *Pkr*, *Isg15*, *Cxcl1*, *Ccl2*, *Ccl5*, *Ifnγ*, *Tlr3*, *Stat1*, *Irf3*, *Irf7*, *Irf9*, and *Ciita*. RNA levels were determined using the 7500 fast real-time PCR system (Applied Biosystems). Gene expression in total tissue mRNA or glial populations was normalized to the respective GAPDH mRNA expression in each mRNA preparation and converted to a linearized value using the formula: 2e(*C_T_*^GAPDH^ − *C_T_*^gene^) × 1,000.

### Immunohistochemistry and immunofluorescence.

Brains from PBS-perfused mice were fixed with 10% neutral zinc-buffered formalin and embedded in paraffin for viral N protein analysis. The distribution of viral antigen (Ag) was determined using MAb J3.3 specific, for the carboxyl terminus of the viral N protein, as the primary MAb, biotinylated horse anti-mouse IgG as the secondary Ab, and streptavidin-conjugated horseradish peroxidase and 3,3′-diaminobenzidine substrate (Vectastain-ABC kit; Vector Laboratories, Burlingame, CA) as described previously (84). High-resolution whole-slide scanning was performed using the Leica SCN400F scanner (Leica Microsystems, Wetzlar, Germany) with a 20× objective. Sections in each experimental group were evaluated, and representative fields were identified.

For immunofluorescence analysis, half brains were embedded in TissueTeck OCT compound (Scigen Scientific, Gardena, CA), flash frozen in liquid nitrogen, and stored at −70°C. Blocks were cut into 12- to 14-μm sections using a cryostat at −17°C. Frozen sections were fixed with 4% paraformaldehyde for 15 min at room temperature and permeabilized in 0.3% Triton-X in PBS for 15 min. Nonspecific Ab binding was blocked using 1% bovine serum albumin, 10% goat or donkey serum, and goat anti-mouse IgG(H+L) (1:300 dilution; Jackson ImmunoResearch, West Grove, PA). Virus-infected cells were identified using MAb J3.3 (31) in combination with cell type-specific markers for microglia/macrophages using rabbit anti-IBA1 (019-19741; Wako, Richmond, VA), astrocytes using rabbit anti-glial fibrillary acidic protein (anti-GFAP) (Dako, Carpinteria, CA), or neurons using rabbit anti-NeuN (ABN78; Millipore, Temecula, CA) Ab. To assess CNS T cell infiltration, sections were stained with rabbit anti-mouse laminin Ab (Cedarlane Laboratories, Ontario, Canada) and rat anti-mouse CD3 MAb (eBioscience). Apoptosis was determined by staining tissue sections for caspase 3 in combination with CD3 (Abcam, Cambridge, MA) or HuR (Santa Cruz Biotechnology, Santa Cruz, CA) to identify T cells and neurons, respectively. Secondary Abs were goat anti-mouse or goat anti-rat Alexa-Fluor 488-, goat anti-rabbit Alexa-Fluor 594-, donkey anti-rabbit Alexa-Fluor 594-, and donkey anti-mouse Alexa-Fluor 488-conjugated IgG (Invitrogen). Sections were mounted with Vectashield antifade mounting medium with 4′,6-diamidino-2-phenylindole (DAPI) (Vector Laboratories, Burlingame, CA). Images were acquired using a Leica TCS-SP5II upright confocal/multiphoton microscope (Leica Microsystems, Wetzlar, Germany). All images were analyzed using Fiji version 1.0. Quantification of viral N protein staining areas and the relative proportion of colocalization with GFAP or IBA1 reactivity were calculated per field of interest using Image-Pro Plus version 7.0. Infected neurons were enumerated by counting entire brain sections. For immunohistological analysis, representative data are presented from 6 or 7 separate fields per mouse with 3 or 4 mice per group per time point.

### IFN-γ ELISA.

IFN-γ in brain supernatants was measured by enzyme-linked immunosorbent assay (ELISA) as described previously (31). Briefly, 96-well plates were coated with 100 μl of 1-μg/ml purified rat anti-mouse IFN-γ MAb (R4-6A2; BD Bioscience) in 0.1 M disodium hydrogen phosphate (pH 9.5) at 4°C overnight. Following blocking with 10% fetal calf serum (FCS) in PBS for 1 h, samples or a recombinant IFN-γ standard (BD Biosciences) was added, and plates were incubated at 4°C overnight. Bound IFN-γ was detected using biotinylated rat anti-mouse IFN-γ MAb (XMG1.2; BD Biosciences) and avidin peroxidase followed by 3,3′,5,5′-tetramethylbenzidine (TMB) (TMB reagent set; BD Biosciences) 30 min later. Optical densities were read at 450 nm with a Bio-Rad model 680 microplate reader and analyzed using Microplate Manager 5.2 software (Bio-Rad Laboratories, Hercules, CA).

### BMDM and stimulation.

Bone marrow-derived macrophages (BMDM) were prepared as described previously (31). Bone marrow was collected from femurs and tibiae of 8- to 10-week-old mice, passed through a cell strainer, and centrifuged at 400 × *g* for 5 min at 4°C. Cells were suspended in BMDM medium (Dulbecco modified Eagle medium [DMEM], 10% FCS, 20% L929 conditioned medium as a source of colony-stimulating factor 1, 0.1% gentamicin, 1% sodium pyruvate) and seeded at a density of 5 × 10^6^ cells in 10 ml BMDM growth medium into 10-mm^2^ tissue culture dishes. An additional 5 ml BMDM medium was added 3 days later. At 7 days postseeding, the fully differentiated and adherent macrophages were washed with DMEM, removed by scraping, and plated into 6-well plates (1 × 10^6^ cells/well). BMDM were confirmed to be >90% F480^+^and CD11b^+^ cells by FACS analysis. Cells were treated with a mixture of poly(I)·poly(C) [poly(I·C)] (5 μg) (high molecular weight; InvivoGen, San Diego, CA) and FuGENE 6 transfection reagent (Promega, Madison, WI) in DMEM following the manufacturer's protocol. After 4 or 12 h of poly(I·C) treatment, poly(I·C) was removed and 50 ng/ml recombinant IFN-γ (BD Pharmingen) was added to cultures and left for 18 h. After stimulation, cells were lysed directly in 800 μl TRIzol (Invitrogen) and stored at −70°C for RNA isolation as described above.

### Statistical analysis.

Statistics were determined using the unpaired two-tailed Student *t* test and two-way analysis of variance (ANOVA) with Bonferroni posttest; the analysis is indicated in the figure legends. Graphs were plotted using GraphPad Prism 5.0 software (GraphPad Software, Inc., LA Jolla, CA).
